# Adenoid cystic carcinoma: insights from molecular characterization and therapeutic advances

**DOI:** 10.1002/mco2.734

**Published:** 2024-09-11

**Authors:** Yunxuan Jia, Yupeng Liu, Haitang Yang, Feng Yao

**Affiliations:** ^1^ Department of Thoracic Surgery Shanghai Chest Hospital Shanghai Jiao Tong University School of Medicine Shanghai China; ^2^ Department of Thoracic Surgery Tumor Hospital Affiliated to Nantong University Nantong Tumor Hospital Nantong China

**Keywords:** adenoid cystic carcinoma, heterogeneity, molecular pathobiology, single‐cell RNA‐sequencing, targeted therapy

## Abstract

Adenoid cystic carcinoma (ACC) is a malignant tumor primarily originating from the salivary glands, capable of affecting multiple organs. Although ACC typically exhibits slow growth, it is notorious for its propensity for neural invasion, local recurrence, and distant metastasis, making it a particularly challenging cancer to treat. The complexity of ACC's histological and molecular features poses significant challenges to current treatment modalities, which often show limited effectiveness. Recent advancements in single‐cell RNA‐sequencing (scRNA‐seq) have begun to unravel unprecedented insights into the heterogeneity and subpopulation diversity within ACC, revealing distinct cellular phenotypes and origins. This review delves into the intricate pathological and molecular characteristics of ACC, focusing on recent therapeutic advancements. We particularly emphasize the insights gained from scRNA‐seq studies that shed light on the cellular landscape of ACC, underscoring its heterogeneity and pathobiology. Moreover, by integrating analyses from public databases, this review proposes novel perspectives for advancing treatment strategies in ACC. This review contributes to the academic understanding of ACC by proposing novel therapeutic approaches informed by cutting‐edge molecular insights, paving the way for more effective, personalized therapeutic approaches for this challenging malignancy.

## Graphical abstract

Adenoid cystic carcinoma exhibits significant cellular heterogeneity, with tumor cells primarily composed of myoepithelial cells and ductal‐like cells. The tumor microenvironment is immunosuppressive, characterized by cancer‐associated fibroblasts, low HLA expression in epithelial cells, and elevated M2 macrophages and myeloid‐derived suppressor cells. Metastatic lesions show increased NOTCH and MYB expression, promoting tumor migration.

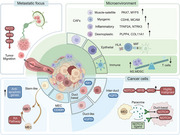

## 1 INTRODUCTION

Adenoid cystic carcinoma (ACC) is a malignant tumor originating from the salivary glands. Since its designation as “cylindroma” by Billroth in 1859, the clinical and pathological characteristics of ACC have gradually been elucidated. ACC is relatively rare, with an annual incidence of 3−4.5 cases per million people, accounting for approximately 1% of all malignant tumors in the head and neck. ACC predominantly originates in the major salivary glands, with the parotid and submandibular glands being the most common sites, accounting for approximately 37% of cases. The remainder arise from minor salivary glands and exocrine glands distributed throughout various organ tissues. Frequently affected areas include the oral cavity (22%), breast (17%), nasal cavity and middle ear (10%), as well as the lungs and bronchi (4%), and pharynx (4%).^1^, ^2^ Furthermore, reported cases extend to the reproductive system, esophagus, and other anatomical locations.^3^, ^4^

Characterized by indolent behavior, ACC exhibits slow growth but is prone to perineural invasion (PNI) and local recurrence. Some cases display notable invasiveness with a propensity for distant metastasis, commonly to the lungs, bones, and liver. The heterogeneity in clinical presentations may be associated with the complex pathological features of ACC. Presently, the pathological diagnosis of ACC involves the identification of three main histological subtypes: cribriform, tubular, and solid patterns. The presence or absence, and the proportion, of the solid pattern are thought to impact patient prognosis.^5^ Contemporary strides in single‐cell RNA‐sequencing (scRNA‐seq) have begun to dissect the extraordinary cellular heterogeneity within ACC tumors, offering fresh perspectives on its biology and potential vulnerabilities.^6^, ^7^

Current treatment options for ACC are quite limited, primarily consisting of radical surgical resection, often followed by adjuvant radiotherapy. Chemotherapy is typically reserved for advanced or metastatic cases, but its efficacy is generally limited.^8^ There is an urgent need for novel therapeutic strategies that can effectively target the molecular underpinnings of ACC. This pressing need for improved treatments drives the focus of our review.

In this review, we first delve into the historical context and clinical characteristics of ACC. We then explore the disease's pathological and molecular features, highlighting the significance of recent scRNA‐seq studies. Subsequently, we assess the current therapeutic landscape, including conventional treatments and innovative approaches. Finally, we integrate these insights to outline future research directions and potential clinical applications, providing a roadmap for advancing ACC management and improving patient outcomes.

## 2 HISTORICAL CONTEXT AND CHALLENGES OF ACC

ACC is historically characterized as a neoplasm with slow progression, yet one that inexorably advances despite therapeutic efforts. Histologically, ACC tumors exhibit several patterns, including cribriform, tubular, and solid forms, each correlating with diverse clinical outcomes. Traditional treatments, primarily surgery and radiation therapy, are limited by ACC's capacity to recur and metastasize, often many years after initial treatment. Chemotherapy, while available, has had restricted success due to the tumor's inherent resistance.

### 2.1 Histopathlogy

#### 2.1.1 Histological subtypes and grading systems

**FIGURE 1:**
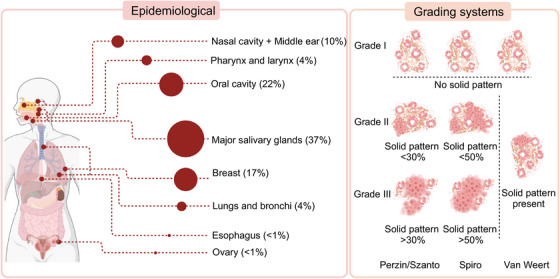
Common sites of occurrence and pathological grading system for ACC. Major salivary glands are the most prevalent sites for ACC, followed by minor salivary glands in the oral cavity, breast, and nasal cavity/middle ear. Distinct pathological grading systems (the Perzin/Szanto, the Spiro, or the Van Weert system). ACC, adenoid cystic carcinoma.

According to WHO guidelines, ACC is a basaloid tumor comprising epithelial and myoepithelial cells, forming distinct patterns with varying prognostic implications.^9^ The cribriform pattern features nests of cuboidal basal epithelial cells with cystic spaces filled with glycosaminoglycans, characterized by alkaline basaloid cells. In contrast, the tubular pattern, associated with the best prognosis, involves ductal structures lined by basaloid cells. The solid pattern, linked to a poorer prognosis, consists of basaloid cells forming sheets within dense fibrous stroma and is associated with higher loss of heterozygosity, chromosomal aberrations, somatic mutations, and increased p53 expression.^10^, ^11^ The proportion of the solid pattern is crucial for ACC grading. The Perzin/Szanto method uses a 30% threshold for the solid pattern to differentiate intermediate from high‐grade ACC, while Spiro suggests 50%.^12^, ^13^ Recent studies propose considering any presence of a solid pattern as an indicator of poor prognosis^14^, ^15^ (Figure 1).

In addition to the three pathological patterns mentioned above, ACC can also manifest a rare pathological feature termed high‐grade transformation (HGT).^16^, ^17^, ^18^ HGT is characterized by highly differentiated ductal and myoepithelial cells, increased mitotic activity, a higher Ki‐67 index, and more p53‐positive cells. HGT‐ACC is more aggressive, with higher risks of local invasion and metastasis.^19^, ^20^, ^21^

#### 2.1.2 Perineural invasion

PNI in ACC often presents as a target‐like arrangement around nerves.^22^ Although its impact on survival is debated, many reports suggest worse disease‐free survival (DFS) and overall survival (OS) for patients with PNI.^23^ PNI significantly increases the risk of local recurrence as ACC can extend along nerves beyond clinically apparent boundaries.^24^ The correlation between PNI and ACC histological patterns is also debated, with some studies indicating higher PNI incidence in cribriform and solid patterns.^25^, ^26^

### 2.2 Immunohistochemistry

Immunohistochemistry is essential in diagnosing ACC and involves key markers that help assess the prognosis and characteristics of the tumor.

#### 2.2.1 Diagnostic markers

Key diagnostic markers for ACC include CD117 (c‐kit) and Ki‐67. CD117 is expressed in up to 90% of ACC tumors and is linked to enhanced invasiveness through mesenchymal transformation.^27^, ^28^, ^29^ Ki‐67 is widely used to evaluate tumor cell proliferation, with higher Ki‐67 indices indicating greater malignancy.^30^ Additionally, markers like minichromosome maintenance protein and proliferating cell nuclear antigen are useful in distinguishing malignant from benign salivary gland tumors, with elevated levels suggesting higher malignancy.^31^, ^32^

#### 2.2.2 Prognostic markers

Prognostic markers in ACC include p53, EphA2, VEGFR, beclin 1, Bcl‐2, and SOX2. p53 is crucial in HGTs and is expressed in nearly half of ACC cases, particularly in the solid histological subtype, where it is associated with increased metastasis, local recurrence, neural invasion, and reduced 5‐year OS.^33^, ^34^ Overexpression of EphA2 and VEGFR is linked to higher micro‐vessel density and perineural infiltration, making them potential prognostic indicators.^35^, ^36^ Beclin 1, related to autophagy, shows that lower expression correlates with poorer histological growth patterns and higher grades.^37^ Bcl‐2, involved in apoptosis, is significantly associated with PNI and worse prognosis.^38^ Meanwhile, SOX2 expression correlates with clinical progression and poor outcomes, while SOX‐10 is broadly expressed in ACC cells, though its link to tumor development is not yet well established.^39^, ^40^, ^41^

## 3 MOLECULAR CHARACTERIZATION: A DOORWAY TO UNDERSTANDING ACC

In addition to the protein‐level immunohistochemical features, ACC displays distinctive genetic traits, typically MYB fusion genes and NOTCH mutations. Moreover, other mutations and copy number variations (CNVs) are intricately linked with ACC. With the advancement of whole‐genome sequencing, more potential targets and new insights into classic targets have been discovered.^42^, ^43^, ^44^ This progress brings new directions for researching the molecular mechanisms and therapeutic strategies of ACC development. Grasping the genetic alterations within the tumor is vital for prognostication and for pinpointing and devising innovative therapeutic strategies.

### 3.1 Genetic alterations

#### 3.1.1 MYB fusion gene

As a recognized specific genetic feature of ACC, the t (6;9) translocation, leading to the MYB–NFIB fusion oncogene, serves as a specific and sensitive marker to distinguish ACC from other salivary gland tumors.^45^, ^46^ The MYB translocation in ACC acts as a driver gene, inhibiting apoptosis via the MYC pathway and promoting cell cycle progression by upregulating CCND1 and downregulating p21.^47^, ^48^ Additionally, MYB facilitates angiogenesis, metastasis, and tumor EMT by upregulating VEGFA, ICAM1, vimentin, N‐cadherin, and α‐SMA, while also regulating apoptosis‐related factors like API5, BCL2, and BIRC3.^49^, ^50^, ^51^, ^52^

Mechanistically, the increased expression resulting from MYB translocation may be linked to super‐enhancers, with the majority of ACC cases exhibiting super‐enhancers in proximity to the MYB gene locus, thereby enhancing its expression.^53^ Moreover, the MYB protein binds to translocated enhancers, and these enhancers, in turn, physically interact with the MYB promoter, driving its expression and establishing a positive feedback loop.^54^ Additionally, MYB overexpression in ACC is associated with 3′ UTR deletion.^55^ Normally, MYB expression is downregulated by miR‐15a/16 or miR‐150. However, in ACC tumor cells, deletion in the 3′ UTR region disrupts its interaction with miRNA, ultimately leading to the overexpression of the *MYB* gene.^51^

**FIGURE 2:**
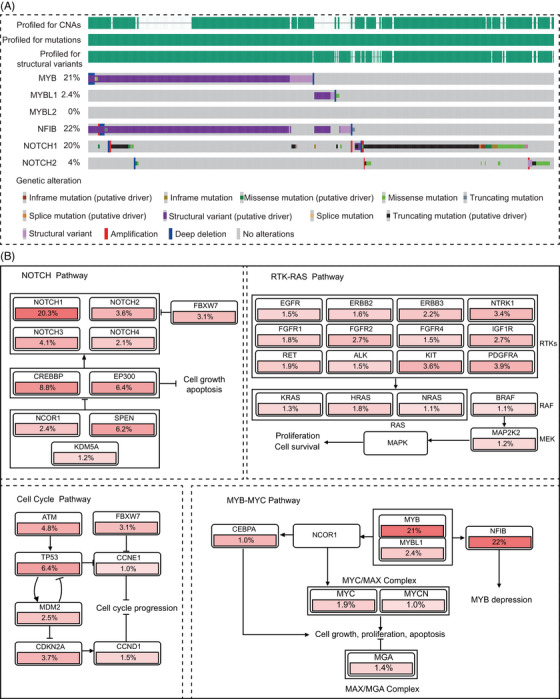
The mutation landscape of ACC. (A) OncoPrint diagram depicting mutations in MYB family and NOTCH family. (B) Enriched pathways in mutated genes of ACC. Data were adapted from public cBioportal (https://www.cbioportal.org/). ACC, adenoid cystic carcinoma.

In addition to the common *MYB–NFIB* fusion, several other fusion genes have gradually gained attention.^54^ Through data mining using the built‐in analysis tools in cBioPortal (https://www.cbioportal.org/), we analyzed the genetic characteristics of 1294 cases of ACC from multiple research projects. The results revealed that nonclassical *MYB* fusions, including *MYB–TGFBR3* and *MYB–RAD51B* fusions, accounted for approximately 2.2% (29 out of 1294) of ACC patients (Figure 2A). Additionally, another member of the MYB transcription factor family, MYBL1 (A‐MYB), also exhibits gene fusions, with a frequency of 2.4% (31 out of 1294), including MYBL1–NFIB and MYBL1–YTHDF3 fusions, whereas no similar phenomena were observed for MYBL2 (B‐MYB). Research indicates that MYBL1 rearrangements can drive ACC development in a manner similar to MYB.^56^ Moreover, there is no significant difference in the gene expression profiles between samples with *MYBL1* fusion genes and those with *MYB* fusion genes.^57^ Besides fusion with the MYB family, NFIB gene fusions, such as NFIB:NFIB self‐fusion, have been identified in some ACC patients, although their impact on ACC remains unclear. Remarkably, our analysis results also indicate that mutations and fusions of the *MYB* and *MYBL1* genes are mutually exclusive, further demonstrating the functional redundancy between these two genes (Figure 2B), attributed to the highly homologous DNA binding domains they possess.^58^

Notably, studies suggest differences in anatomical location and patient prognosis between *MYB* and *MYBL1* fusion genes. Interestingly, *MYBL1* fusion genes/overexpression tend to occur between the submandibular regions.^56^ Compared with salivary ACC patients with *MYBL1* alterations, those with *MYB* alterations have shorter survival. In multivariate analysis, the 3′ deletion of *MYB* is an independent prognostic marker for OS, significantly enriched in grade III tumors (solid pattern > 30%).^59^ This seemingly contradictory conclusion may be related to the expression location of *MYBL1*. Mitani et al.^56^ speculated that MYBL1 may be actively expressed in specific cells in the submandibular region, and due to the loose arrangement of this gene locus, it is prone to physical DNA damage and misrepair.^60^ Unlike MYB, which is expressed in various tissues throughout the body (e.g., colorectal cancer and breast cancer), the expression of MYBL1 is limited to developing breast tissue, testicular germ tissue, the central nervous system, and T and B lymphocytes.^61^ This differential expression in different cells may explain the differences in the impact of MYB and MYBL1 on patient prognosis, despite having common downstream effects.

Currently, MYB has emerged not only as a diagnostic marker but also as a significant therapeutic target for ACC. Clinical trials employing MYB inhibitors to target ACC tumor cells are underway, offering a promising direction for new drug development.

#### 3.1.2 NOTCH mutations

NOTCH proteins, as heterodimeric transmembrane receptors, play a crucial role in various cellular processes, including differentiation and proliferation, contributing to tissue homeostasis. Comprising transmembrane and extracellular subunits, NOTCH receptors interact with transmembrane ligands (DLL, Jagged, DLK) from neighboring cells. Upon binding, the transmembrane subunit undergoes proteolytic cleavage, releasing the NOTCH intracellular domain (NICD).^62^ NICD is a transcription factor that, upon binding to RBPjκ, regulates the expression of target genes such as *HES1* and *REST*, influencing various cellular events including apoptosis, EMT, migration, and angiogenesis.^63^, ^64^ Its noncanonical pathway also participates in the gene expression of various signaling networks, such as PI3K–mTORC2–AKT and NF‐κB.^65^, ^66^

The role of NOTCH mutations in ACC is gradually being elucidated. In our data analysis, *NOTCH1* mutations and structural variations account for 20%, representing the second‐largest genetic feature in ACC after MYB. This suggests its potential role as a driving gene in ACC.^42^, ^67^ NOTCH is involved in cell proliferation and angiogenesis in ACC through the MAPK pathway, but also in intercellular communication and activation of cascade reactions leading to differentiation of cell populations.^68^, ^69^ Moreover, dysregulation of NOTCH1 can induce EMT by regulating MMPs, thus participating in ACC tumor invasion and metastasis processes.^64^

#### 3.1.3 Other mutations

RAS missense mutations are considered risk factors for DFS and OS.^70^, ^71^ *TP53* mutations correlate with the expected OS of recurrent and metastatic ACC, and they are associated with histological subtypes, with a higher prevalence in the solid histological subtype.^34^, ^72^ Ferrarotto et al.’s^42^ multiomics data also identified a subset characterized by high P63 expression. Activation of TP63 is associated with a better prognosis.^73^ Evidence suggests that TP63 activation typically correlates with lower levels of the NOTCH/MYC pathway and can upregulate and drive the expression of receptor tyrosine kinases such as AXL, epidermal growth factor receptor (EGFR), and MET.^69^, ^74^ Therefore, TP63 may be positively correlated with sensitivity to tyrosine kinase inhibitors (TKIs).

For common mutations, survival analysis was conducted on 196 patients with available survival data. The results indicated that *NOTCH1* (HR = 2.491, 95%CI = 1.338–4.636, *p* < 0.001) and *KDM6A* (HR = 3.428, 95%CI = 0.89–11.890, *p* = 0.0012) significantly impacted patient OS. NOTCH1 is a well‐recognized prognostic marker, while KDM6A, as a histone demethylase involved in the demethylation of H3K27me1‐3 sites of super‐enhancers, may offer new insights into ACC treatment. Considering the 10 TCGA pan‐cancer pathways, it is noteworthy that except for pathways such as NOTCH, PI3K, and RTK–RAS, for which targeted drugs have been developed and play a crucial role in ACC, there is relatively less research on the HIPPO–YAP pathway. Key points in the HIPPO–YAP pathway, such as *FAT1* and *FAT3*, are frequently mutated in ACC, and *YAP1* is a common mutation site in lung ACC. Chen et al.’s study suggested that key targets in the HIPPO–YAP pathway play a significant role in the biological behavior of ACC, indicating a potential research direction for the next generation of targeted drugs.^75^

#### 3.1.4 Genomic instability and CNVs

Additionally, epigenetic changes are of significant importance. Gene mutations related to chromatin remodeling, such as *SMARCA4, SMARCB1, SMARCC1, SMARCC2, ARID1*, and *PBRM1*, resulting in gene downregulation, have been reported.^67^, ^76^, ^77^, ^78^ Among these mutations, mutations in genes encoding subunits of the SWI/SNF chromatin remodeling complex are particularly crucial. These mutations lead to an imbalance in the expression of SWI/SNF subunits, affecting chromatin accessibility and subsequently regulating the expression of downstream genes, potentially leading to chemotherapy resistance.^79^, ^80^

**FIGURE 3:**
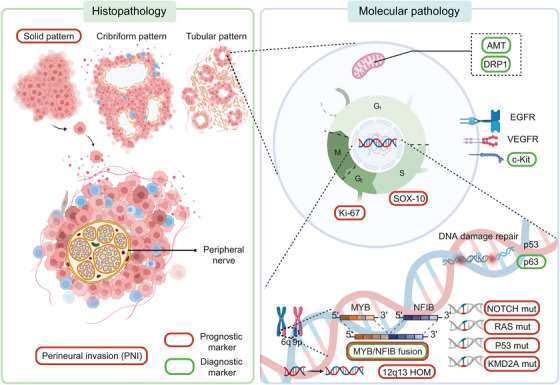
Representative histopathology and molecular pathology features in ACC. Green boxes highlight diagnostic markers, including mitochondrial markers (AMT, DRP1), and membrane receptor c‐Kit. Red boxes represent prognostic markers, such as PNI, solid pattern proportion, and molecular indicators including Ki‐67, SOX‐10, NOTCH, RAS, P53 mutations, KMD2A, and increased copy numbers of 12q13. The MYB: NFIB fusion gene serves as both a diagnostic hallmark and a prognostic factor. ACC, adenoid cystic carcinoma; PNI, perineural invasion.

Regarding CNVs through data mining in cBioPortal, the most common occurrences were 4q12 Amp (3.9%) and 9p21.3 HomDel (3.7%), but the CNV with a significant impact on prognosis was 12q13.11 and 12q13.12 HOMDEL (HR = 3.082, 95%CI = 0.564–16.855, *p* = 0.0206). However, due to the multitude of genes on the 12q13.11‐12 sub‐band, with many genes’ roles in tumors undefined, enrichment was observed only in the WNT pathway (*WNT1*), suggesting its potential as a prognostic target in ACC. The intricate diagnosis and prognostic markers of ACC are summarized in Figure 3.

### 3.2 Molecular pathways involved in ACC

#### 3.2.1 NOTCH–MYC/HEY pathways

The expression of the NOTCH family and its downstream targets can serve as references for the diagnosis and treatment of ACC. As the most important direct downstream target of the NOTCH family, MYC plays a critical driving role in ACC tumorigenesis. Ferrarotto et al. used whole‐transcriptome RNA‐seq and targeted RPPA proteomics to cluster ACC patients into two groups.^42^ Among these, the ACC‐I class, characterized by NOTCH–MYC pathway activation, had a significantly worse prognosis and was associated with solid components, minor salivary gland origin, and mutations in SPEN, CREBPP, and EP300. Additionally, resistance to NOTCH inhibitors (GSIs) is linked to the re‐expression of MYC through non‐NOTCH mechanisms, such as the use of alternative enhancers, indicating that MYC can be a crucial drug target within the NOTCH pathway.^68^, ^81^, ^82^ Furthermore, RNA‐seq results from Xie et al.^64^ indicate that, besides HES1 and MYC, HEY is a major downstream effector of NOTCH1 in ACC. These genes act as oncogenes in ACC, accelerating the proliferation and migration of ACC cells. The NOTCH1–HEY1 pathway is particularly upregulated in ACC, promoting EMT and the expression of various MMP family members, thereby playing a vital role in the proliferation, invasion, metastasis, and apoptosis of ACC tumors.^64^, ^83^

In summary, the NOTCH signaling pathway serves as a crucial differentiation signal for tumor cells, while MYB acts as a marker for undifferentiated stem cells by inhibiting differentiation programs and promoting stem cell proliferation. Therefore, NOTCH1 and MYB have opposing effects on ACC differentiation. This is partially supported by our integrated data, showing a mutually exclusive pattern of expression between the MYB family and NOTCH1 (Figure 2A). Studies have shown that patients with such mutations often experience faster tumor development, and the incidence of distant metastases such as bone metastasis and liver metastasis is also higher compared with the wild‐type population.^84^, ^85^, ^86^, ^87^

#### 3.2.2 EGFR–PI3K–AKT/MEK–ERK pathways

The EGFR family, through the PI3K–AKT or MEK‐ERK pathways, participates in the proliferation and migration of tumor cells (Figure 2B).^88^, ^89^ In ACC patients, the mutation rate is approximately 1−2%. Extensive literature has reported a correlation between EGFR overexpression and poor prognosis.^42^, ^90^ In addition to its association with mechanisms related to metastasis and proliferation, such as EMT, research suggests that EGFR activation also induces the expression of PD‐L1 through c‐Myc.^91^ Liu et al.^40^ demonstrated that simultaneous targeting inhibition of c‐Myc and EGFR signaling pathways effectively enhances therapeutic potential, indicating that c‐Myc may be a potential target for EGFR pathway resistance.

### 3.3 Molecular insights into ACC histopathology

The pathological subtypes of ACC are associated with numerous molecular targets. Copy number losses at certain loci have been proven to be related to the solid pattern and higher histological grades. Multiomics analysis by Persson et al.^92^ revealed a significant association between copy number loss in 1p36 and a high proportion of the solid pattern. Downregulated genes such as *TP73, MFN2, KIF1B, DFFA*, and *DFFB*, primarily related to apoptosis, were identified in this context.^93^ Additionally, the significant decrease in the expression of the tumor suppressor gene *PARK2* due to 6q loss was associated with a higher histological grade.^92^ Another study suggested that translocations involving MYB or MYBL1 and their high expression, especially the 3′part loss of MYB, were also associated with a high proportion of the solid pattern.^59^

A more systematic study showed that compared with grade I and II ACC, grade III tumors had a significantly higher average number of CNVs per tumor.^94^ Specifically, the loss of 14q was associated with lower histological grades, occurring only in grade I tumors, while losses of 1p, 6q, and 15q, along with nonrecurrent gains/losses of the whole chromosome, were associated with higher‐grade tumors. Moreover, genes in the 1p36.33‐p35.3 locus (such as *CTNNBIP1*, *CASP9*, *PRDM2*, and *SFN*) may have potential pathogenic roles or guiding significance for patient treatment and prognosis in higher‐grade tumors.^95^ Some immunohistochemical markers, such as Ki‐67 and p53 indices, have been proven to be related to higher‐grade components. The relationship between the Ki‐67 index and tumor tissue subtypes remains debated. No correlation was observed in the study of tracheobronchial ACC pathology characteristics^96^; however, in another study with a larger sample size focusing on minor salivary gland ACC, Ki‐67 immunoreactivity increased with the increase in ACC histopathological grade.^97^, ^98^

## 4 scRNA‐seq: A NEW FRONTIER IN ACC RESEARCH

The advent of scRNA‐seq technologies has revolutionized our understanding of the heterogeneity within ACC tumors. By analyzing gene expression at the single‐cell level, researchers have now begun to categorize distinct cellular subpopulations within the tumors, encompassing cancer cells, immune cells, fibroblasts, and endothelial cells, each contributing to the tumor's biology and therapeutic resistances.

### 4.1 Cancer cell heterogeneity

ACC tumor tissue harbors two distinct malignant cell populations resembling myoepithelial cells and duct‐like basal cells of normal salivary glands. However, the origin and functions of these two cell types remain inconclusive. Viragova et al.^7^ purified and separated these two cell populations using CD49f (myoepithelial cell marker) and KIT (duct‐like cells marker). They found that these cells differentiated from different epigenetic lineages rather than accumulating somatic mutations. Despite the lower proliferation rate of myoepithelial‐like cells, they represent a highly tumorigenic component in ACC, supporting tumor formation in patient‐derived xenograft (PDX) models. Additionally, myoepithelial‐like cells have been identified as progenitor cells for ductal‐like cells, and their differentiation into ductal‐like cells can be promoted by the activation of RA signaling.^7^ This finding explains the poor efficacy of all‐trans retinoic acid (RA) (ATRA): the activation of RA signaling merely disturbs the proportional balance between myoepithelial‐like cells and ductal‐like cells within ACC tumors. On the other hand, the RA signaling inhibitor BMS493 can suppress tumor development by inducing selective death of ductal‐like cells, offering new insights for ACC treatment.

As mentioned earlier, ACC is predominantly characterized by genetic alterations of the MYB family and NOTCH family. Moreover, genetic alterations of the MYB family and NOTCH family are mutually exclusive (Figure 2B). Interestingly, Parikh et al.^99^ also identified heterogeneity in MYB and NOTCH expression in these two cell types, with higher NOTCH and its target gene expression in duct‐like cells. Notably, NOTCH ligands were predominantly expressed by myoepithelial cells, indicating a potential paracrine signaling system between the two subpopulations.^99^ Additionally, cell clusters with high MYB expression also exhibit elevated expression of multiple genes associated with tumor invasion and metastasis, particularly NTRK3, which encodes a member of the neurotrophic tyrosine receptor kinase family.^100^ This suggests that the function of these cells may be related to neural pathways, consistent with the clinical characteristics of ACC, including its growth and neural infiltration features.

Lin et al.^6^ further subdivided duct‐like cells populations into intercalated ductal‐like cells (KRT19+/AQP5+/KIT+) and duct‐like cells (KRT19+/AQP5−/KIT−), with intercalated ductal‐like cells showing direct relevance to tumor development, participating in NOTCH‐ and MYC‐signaling‐driven tumor proliferation and differentiation pathways, and involvement in immune responses. Zhou et al.^84^ identified a subpopulation of cancer stem cells with high expression of apoptosis resistance genes, suggesting their role in metastasis and relapse.

The cellular landscape among ACC tissue subtypes also varies. In solid patterns, duct‐like cells markers are significantly upregulated, and myoepithelial cells exhibit greater variability, with notable upregulation of KRT14 and KRT16P3 compared with other patterns. In higher‐grade transformed ACC tissues, myoepithelial cells are nearly absent, and the significant activation of the NOTCH signaling pathway, possibly no longer dependent on paracrine signals from myoepithelial cells, is associated with *NOTCH1* mutations.^99^

### 4.2 Tumor microenvironment landscape

The tumor microenvironment (TME), including metabolic and immune components, is closely associated with tumor behavior. Clustering analyses indicate the presence of nontumor cells in the ACC TME, such as tumor‐associated fibroblasts (CAFs), immune cells, and endothelial cells. The proportions are epithelial cells (40.1%), fibroblasts (27.9%), T/NK cells (11.3%), and endothelial cells (10.8%).

Parikh et al.^99^ categorized ACC CAFs into proliferative CAFs expressing muscle satellite cell markers (PAX7, MYF5), myogenic CAFs expressing pericyte markers (CDH6, MCAM), inflammatory CAFs expressing EMT markers (TFAP2A, NTRK3), and fibrotic CAFs expressing adipose stromal cell markers (PLPP4, COL11A1).

ACC exhibits a low mutation load, with reduced tumor antigen availability and presentation capabilities, and is characterized by an immunosuppressive microenvironment compared with other salivary gland tumors. Studies indicate that, compared with normal tissues, tumor‐infiltrating lymphocytes are significantly reduced in ACC. Cytotoxic CD8+ T cells and CD4+ T helper cells are notably downregulated, and IgA plasma cells, which regulate mucosal immunity, are completely absent.^100^, ^101^ Additionally, the PD1/PDL1 signaling pathway is significantly inhibited, while immunosuppressive PD‐L2 signaling and HLA‐G are activated, explaining the generally poor response of ACC to immunotherapy.^102^ The primary immune cells in ACC include two clusters of macrophages: one comprising inflammatory macrophages (IL‐1B), and the other associated with EMT. In normal tissues, macrophages primarily participate in antigen presentation via MHC‐II and in the interaction between various cells. However, in ACC tumors, macrophages’ functions are mainly related to neutrophil activation and the initiation of inflammation.^100^

Despite ACC's generally immunosuppressive nature, cell communication between macrophages and T cells or epithelial cells is typically highly upregulated. In normal tissues, immune cell–epithelial cell communication is mainly mediated by HLA–MHC, but this pattern is significantly downregulated in the ACC TME, replaced by the macrophage migration inhibitory factor (MIF)‐CD74 signaling pathway.^6^ Research suggests that MIF‐CD74 enhances tumor proliferation by promoting angiogenesis in the TME and inhibiting tumor cell apoptosis.^100^ MIF might also serve as a new prognostic marker for ACC.

### 4.3 Paracarcinoma and metastatic lesion cellular landscape

In comparison with primary ACC lesions, metastatic ACC lesions exhibit elevated MYB expression, and metastatic cell lines show higher MYB expression but lower NOTCH1 expression than indolent cell lines. Mechanistically, metastatic cell lines recruit MYB via NICD to activate MYC, ultimately promoting tumor migration. Lymph node metastases do not show consistent expression differences compared with primary lesions, suggesting their role primarily as conduits for cell migration without significant biological function.^84^ Recurrent ACC exhibits increased NOTCH signaling and reduced MYB expression, undergoes a transformation in cellular composition, and displays distinct clustering compared with primary ACC, with a tendency to differentiate preferentially toward duct‐like cells.^99^

In clinical practice involving ACC surgeries, positive tumor margins that are not visible to the naked eyes are often encountered.^103^, ^104^ This could be related to the oncogenic activity within the paracarcinoma tissue. Lin et al.^6^ discovered complex CNVs in the epithelial cells of ACC paracarcinoma tissue, leading to the identification of a significant population of pre‐M cells, constituting approximately 47% of the epithelial cells in the paracarcinoma tissue. These pre‐M cells also exhibit high expression of characteristic ACC genes such as MYB, MYBL1, and EN1. Their stem cell scores are higher than those of tumor cells, and pseudotime analysis indicates their potential to differentiate into tumor cells. This finding may provide new insights into the molecular mechanisms underlying ACC tumor recurrence.^6^

Despite the advancements in scRNA‐seq, which have allowed researchers to depict the cellular heterogeneity of ACC and partly explain its high local recurrence rate and response to certain drugs (e.g., RA agonists), there are still significant gaps. Studies by Parikh, Zhou, and Viragova have all focused on the effects of the RA pathway on the differentiation and paracrine signaling of ACC cell subpopulations, but they have reached contradictory conclusions. The impact of RA pathway inhibitors and agonists on ACC cell subpopulations requires further exploration. Lin et al.’s research is the most comprehensive, identifying different subtypes of ductal epithelial cells and a cluster of precancerous cells.^6^ However, this study fails to define the functions of different cell subtypes and explore their therapeutic targets. Current scRNA‐seq studies on ACC are mainly limited to transcriptomics, with no research on metabolomics and spatial gene distribution. Additionally, the functions and characteristics of different cell subpopulations are mostly based on superficial analysis of sequencing results, without experimental validation of their intrinsic molecular pathways. Therefore, there remains significant research potential in this field (Table 1).

**TABLE 1:** Summary of scRNA‐seq sequencing studies in ACC.

Authors	Sample size	Anatomical site	Year	Main outcomes	References
Lin et al.	3	Major salivary glands	2022	Identified intercalated ductal‐like cells involved in tumor development and NOTCH /MYC signaling Defined precancerous epithelial cells (pre‐M) with higher stem cell scores, linked to local recurrence	^6^
Zhou et al.	5	Major salivary glands	2022	ATRA can activate NOTCH1 and inhibit MYB expression, counteracting the NOTCH1–MYB–MYC axis to suppress lung metastasis in ACC. Identified a subpopulation of cancer stem cells with high apoptosis resistance gene expression, indicating a role in metastasis and relapse	^84^
Viragova et al.	7	Oral cavity,; parotid gland, and trachea	2023	Myoepithelial‐like cells show higher tumorigenicity compared with duct‐like cells and act as progenitor cells. RA inhibitors selectively target ductal‐like cells and inhibit tumor growth in ACC PDX models.	^105^
Parikh et al.	7	Major salivary glands	2022	Classified ACC CAFs into proliferative, myogenic, inflammatory, and fibrotic subtypes based on specific markers Identified heterogeneity in MYB and NOTCH expression, with higher NOTCH and target gene expression in duct‐like cells and NOTCH ligands mainly in myoepithelial cells	^99^
An et al.	1	Submandibular gland	2023	TME mainly consists of two macrophage clusters: inflammatory (IL‐1B) and EMT related. Enhanced cell communication in tumor tissue, with MIF‐CD74 and APP‐CD74 significantly upregulated	^100^

Abbreviations: ATRA, all‐trans retinoic acid; TME, tumor microenvironment; ACC, adenoid cystic carcinoma; PDX, patient‐derived xenograft; CAFs, cancer‐associated fibroblasts.

**FIGURE 4:**
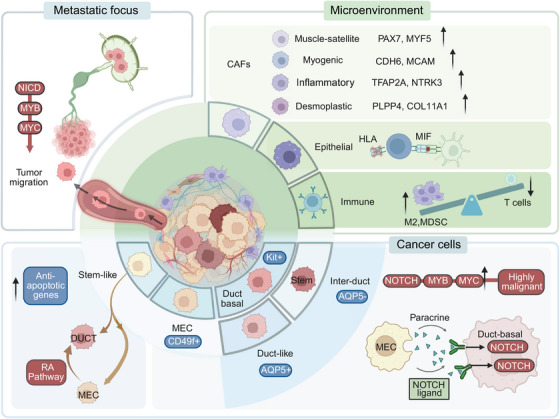
Cellular landscape of ACC. ACC tumor cells exhibit significant heterogeneity, primarily composed of MECs and DUCTs. MECs show high expression of NOTCH ligands, engaging in paracrine signaling with DUCTs expressing high levels of NOTCH receptors, facilitating tumor cell proliferation and differentiation. Moreover, DUCTs can be further categorized into ductal‐like cells and intercalated ductal‐like cells, with the latter exhibiting high NOTCH signaling and increased oncogenic potential. The tumor microenvironment consists of CAFs, epithelial cells, and immune cells. CAFs display strong heterogeneity, including subgroups like myoepithelial cells and myofibroblasts, as well as subsets involved in inflammation and desmoplasia, each with distinct molecular markers. Epithelial cells exhibit low HLA expression, primarily communicating with macrophages or T cells through MIF. ACC tumor antigen presentation and T cell activation are notably low, while immune‐suppressive cells like M2 macrophages and MDSCs are elevated, creating an overall immunosuppressive environment. Compared with primary lesions, metastatic lesions show significantly higher expression of NOTCH and MYB, with metastatic cell lines recruiting MYB through NICD to activate MYC, ultimately promoting tumor migration. In contrast, lymph node metastases exhibit no significant differences from primary lesions. ACC, adenoid cystic carcinoma; CAFs, cancer‐associated fibroblasts; DUCTs, ductal‐like cells; MDSCs, myeloid‐derived suppressor cells; MECs, myoepithelial cells.

Collectively, the molecular underpinnings of ACC are as diverse as its clinical presentation (Figure 4). Until recently, investigations into the genomic drivers of ACC have been hindered by this heterogeneity. The identification of the MYB–NFIB fusion gene in a subset of ACC cases was a landmark discovery. Furthermore, alterations in genes such as TGF‐β, NOTCH, and PI3K have been implicated in ACC progression. Yet, this molecular understanding did not fully explain the varying behaviors and responses to therapy observed among patients. ScRNA‐seq studies have revealed not only the phenotypic plasticity of ACC tumor cells, which can dynamically switch states, but also the complex interplay between these cells and the TME. This dialogue, mediated through a vast network of cytokines, growth factors, and extracellular matrix components, is thought to play a critical role in ACC's invasive capacity and resilience against conventional therapies. Therefore, The detailed exploration of ACC's histological and molecular features sets the stage for understanding the challenges and opportunities in current therapeutic strategies.

## 5 THERAPEUTIC ADVANCES: SHIFTING THE PARADIGM IN ACC TREATMENT

### 5.1 Surgical approaches

The primary treatment for ACC involves surgery, regardless of the tumor's location, with complete surgical resection being the preferred approach. The surgical technique varies depending on the ACC's site, but the main challenge is preserving critical nerves and other vital structures while striving to achieve clear margins.^11^, ^106^

### 5.2 Radiation therapy

Because achieving clear margins in surgery is difficult, postoperative radiation therapy (PORT) is widely used for advanced ACC.^107^, ^108^, ^109^ Studies show that PORT improves survival rates in high‐grade and locally advanced ACC.^110^, ^111^, ^112^ Thus, PORT is recommended for all ACC patients except those with T1N0 lesions and negative margins.^113^, ^114^ However, some research suggests that while PORT may enhance quality of life, it does not significantly impact survival rates. For patients with lymph node metastasis, radiation therapy improves prognosis, while it is not recommended for those without lymph node involvement.^115^ Radiation modalities for ACC include photon therapy, carbon ions, protons, and neutrons, though clear guidelines for choosing between these modalities are currently lacking.^116^

#### 5.2.1 Proton therapy

Protons, characterized by low linear energy transfer (LET), offer superior depth dose distribution, enabling higher radiation doses to tumors while minimizing exposure to healthy tissues.^117^ Intensity‐modulated proton therapy (IMPT) studies by Pommier demonstrated increased total doses (70.0–79.1 CGE), significantly enhancing local control rates without neurological toxicity.^118^, ^119^, ^120^

#### 5.2.2 Neutron therapy

Unlike conventional photon therapy, which treats tumors by forming free radicals and reactive oxygen species, fast neutron radiotherapy primarily relies on its high LET rate. This high LET results in more unrepairable double‐stranded DNA breaks within cells, maintaining good radiation sensitivity even in ACC tumors with low metabolic activity.^121^ Consequently, it minimizes cell damage repair and has a lower oxygen enhancement ratio (OER).^122^ Clinical trials have shown that, compared with photon therapy, fast neutron radiotherapy provides higher short‐term and long‐term local control rates and survival rates in advanced ACC cases.^123^ It is also effective for patients resistant to conventional photon therapy.^124^

In contrast, boron neutron capture therapy (BNCT) exerts its cytotoxic effects through an in situ nuclear reaction between boron atoms, injected into the tumor site, and low‐energy neutrons.^125^, ^126^ This approach not only significantly increases the bioavailability of the treatment but also markedly reduces damage to surrounding tissues. BNCT has shown preliminary applications in radiotherapy for ACC located in critical areas such as near the base of the skull.^127^, ^128^

#### 5.2.3 Carbon ion therapy

Carbon ions, charged heavy particles, enable precise tumor targeting with minimal impact on surrounding tissues.^129^, ^130^ Phase I/II trials demonstrated significantly higher local control rates than conventional therapies. Pencil beam scanning–carbon ion radiotherapy (CIRT) in ACC treatment achieves conformal dose distribution similar to intensity‐modulated radiation therapy, improving local control and reducing adverse effects.^130^, ^131^, ^132^, ^133^ CIRT exhibits the lowest OER, which is the ratio of doses necessary to inactivate tumor cells in hypoxic and oxic conditions. It is a powerful tool for overcoming tumor hypoxia, reducing the OER value to 1.0. The efficacy of carbon ion therapy is 1.5–3 times higher than photon therapy due to its superior relative biological effectiveness.^134^

No unified guideline exists for selecting radiation types. Alexander outlined pros and cons, suggesting IMPT and low‐dose CIRT minimally impact normal organs, potentially better controlling local progression.^135^ Huber et al.^136^ recommended neutron therapy for patients with poor prognosis, large residual lesions, or unresectable tumors. In summary, the choice between photon, proton, or carbon ion irradiation depends on individual cases and treatment goals. Proton therapy, particularly IMPT, and carbon ion therapy have shown promise in improving local control with minimal toxicity, while neutron therapy may benefit patients with specific clinical characteristics (Table 2). Research continues to refine treatment guidelines and explore novel approaches for ACC management.

**TABLE 2:** Characteristics of different radiotherapy modalities.

Radiation types	LET	Effectiveness	Physical dose distribution	OER	Cost
Photon^137^	Low	/	Gradual dose decrease with depth	2.5–3	Low
Proton^138^	Low	Less damage to the normal tissue	Target conformality with a Bragg peak	2.5−3	Medium
Carbon Ion^129^	High	Superior local tumor control	Target conformality with a sharper Bragg peak	1.0	High
Neutron^122^, ^139^	Medium	Strong biological impact, damaging normal tissues	Complex dose profile	1.3–1.7	High

Abbreviations: LET, linear energy transfer; OER, oxygen enhancement ratio.

Additionally, given the distinct advantages and disadvantages of different radiation types, combining various radiation therapies has emerged as a new direction in ACC radiotherapy. While fast neutron therapy has a high LET, it may not achieve effective doses for tumors near the skull base due to toxicity concerns. Aljabab et al.^124^ achieved good results by combining proton beam therapy with fast neutron therapy for such ACC patients. Similarly, Postuma et al.^140^ found success using BNCT combined with CIRT in patients with locally recurrent ACC. Therefore, exploring how to adjust the doses of different combined radiation components to effectively kill tumors while minimizing toxicity may be a feasible approach for ACC treatment.

### 5.3 Systemic therapies

Chemotherapy, as a traditional treatment for ACC, primarily relies on platinum‐based drugs such as cisplatin combined with paclitaxel. However, current clinical trials report limited effectiveness, with response rates ranging from only 15 to 25%.^141^, ^142^, ^143^ Consequently, the focus of systemic therapy has shifted toward targeting specific molecular pathways of ACC to achieve better tumor control.

#### 5.3.1 Targeted therapy

*MYB inhibitors*. In recent years, clinical studies have illuminated the correlation between ATRA and MYB targets. This medication has been observed to reduce MYB binding in the enhanced region of patient xenograft models harboring MYB translocation. As a result, it attenuates the positive feedback loop associated with MYB overexpression cycling, ultimately leading to a reduction in tumor proliferation.^84^ Currently, two ongoing clinical trials (NCT03999684, NCT04433169) aim to explore the efficacy of ATRA in treating advanced ACC patients.^144^ Additionally, a study investigating the use of a MYB vaccine in combination with a novel anti‐PD‐1 therapy (NCT03287427) is also underway.^145^ RGT‐61159 is an innovative oral drug that selectively modulates RNA splicing to inhibit the expression of the oncogenic transcription factor c‐MYB. Preclinical studies in rodents and nonhuman primates have shown that RGT‐61159 effectively suppresses c‐MYB production and tumor growth in various ACC PDX models. Currently, a phase I clinical trial is underway to further investigate its safety and efficacy.^146^

Some in vitro analyses have identified novel MYB inhibitors that show promise for application.^147^ Yusenko et al.^148^ screened a library of 1280 approved small molecules and found that the polyether ionophore Monensin A exhibited potent MYB inhibitory effects. Additionally, Bcr‐TMP and the proteasome inhibitor oprozomib have been demonstrated to inhibit MYB's oncogenic activity in a p300‐dependent manner, offering the potential for further development as novel MYB inhibitors, pending confirmation through clinical trials.^149^, ^150^

**FIGURE 5:**
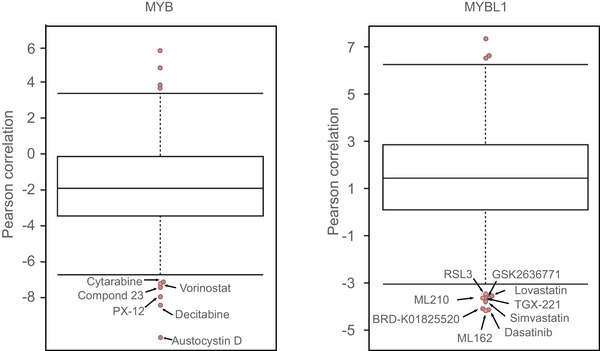
Boxplots illustrating the drug compounds whose expression exhibits a significant correlation with the expression of *MYB* or *MYBL1*. Box‐and‐whisker plots illustrate the correlation between the cytotoxic effects (reflected by the area under the curve [AUC] value) of drug compounds in the library (*n* = 481) and the expression levels of genes (https://portals.broadinstitute.org/ctrp.v2.1/?page=#ctd2Dataset). Note that cells with smaller AUC values are more sensitive to the tested drugs. Several inhibitors were highlighted as having the most negative correlations, indicating that higher expression of MYB/MYBL1 significantly correlates with increased sensitivity to these inhibitors.

Due to the unique role of MYB in the development of ACC, drug development targeting this site holds the most promise for ACC treatment. By analyzing the correlation between the expression levels of *MYB* or *MYBL1* and the drug sensitivity profiles of 481 small molecules across all adherent cell lines in the CTRP portal, we found that among drugs exhibiting a negative correlation with MYBL1 expression levels, GPX4 inhibitors ML162, ML210, and 1S,3R‐RSL‐3, known to induce ferroptosis,^151^ ranked 1st, 7th, and 9th, respectively (Figure 5). Among drugs correlated with MYB expression levels, the pan‐HDAC inhibitor vorinostat ranked 5th. HDAC inhibitors are classical super‐enhancer suppressors, reminiscent of the typical functions of MYB.^53^ Additionally, HDAC can regulate cellular iron homeostasis and the sensitivity to ferroptosis.^152^, ^153^, ^154^, ^155^ All these findings suggest that the iron death mechanism triggered by HDAC inhibitors may play a crucial role in ACC treatment.

*TKIs*. Currently, TKIs targeting c‐kit and EGFR are the main focus of clinical trials for ACC treatment. Imatinib was the earliest TKI applied to ACC, with six studies evaluating its monotherapy effects, reporting only two objective responses in 71 evaluable patients, lasting 14 months and at least 15 months.^156^ Another trial evaluated the efficacy of combining imatinib with cisplatin, where 17 assessable patients only showed three partial responses.^157^, ^158^ The c‐kit‐targeting TKI dasatinib also demonstrated a low objective response rate of 2.5% (one out of 20).^159^ Trials with EGFR inhibitors gefitinib (19 patients) and cetuximab (23 patients) did not show objective responses, but some patients experienced stable disease and extended OS.^160^, ^161^ Lapatinib, a dual inhibitor of EGFR and HER2, did not produce objective responses in 19 subjects.^162^

As previously mentioned, in ACC patients with low NOTCH/MYC expression, TP63 activation induces the expression of receptor tyrosine kinases such as AXL, EGFR, and MET. This makes multikinase inhibitors targeting AXL, MET, and VEGFR, such as cabozantinib, a potential treatment method, currently undergoing clinical trials (NCT03729297).^163^ Additionally, studies have shown that AXL and MET mediate resistance to EGFR inhibitors in certain solid tumors, suggesting that combining cabozantinib with EGFR inhibitors could be a promising therapeutic strategy.^164^

A multicenter phase II study showed that lenvatinib, a triple TKI targeting VEGFR, FGFR, and PDGFR, has a high disease control rate (DCR) and stability in most recurrent or metastatic ACC cases.^165^ Out of 32 patients, 13 (40.6%) experienced clinical benefits for 6 months or longer. An early‐phase trial with the nonselective VEGFR inhibitor axitinib (NCT03990571) found four partial responses in 28 patients (ORR = 18%).^81^ Another phase II study with the multitarget inhibitor sorafenib reported an objective response rate of only around 10%, and adverse reactions were frequent.^166^, ^167^ Moreover, a selective VEGFR2 inhibitor, apatinib, showed promising clinical efficacy with a response rate of 92.3% in patients with recurrent or metastatic ACC, as observed in NCT02775370, and another international multicenter phase II trial reported an objective response rate of 15.3% and a median duration of 14.9 months, both superior to other TKIs and chemotherapy drugs.^168^, ^169^

*NOTCH inhibitors*. A phase I trial (OMP‐52M51) evaluated the efficacy of the monoclonal antibody brontictuzumab, a humanized monoclonal antibody targeting the NOTCH1 protein, in solid tumor experiments. Among 12 ACC patients, two showed partial responses and three experienced stable disease.^170^ Given the close association between the NOTCH pathway and cancer stemness, another phase Ib/II study explored the treatment of ACC patients with the cancer stemness kinase inhibitor amcasertib (BBI503).^171^ Preliminary results indicated an 86% DCR, with a median OS of 28.3 months.

Additionally, γ‐secretase inhibitors act in the intracellular domain to participate in the cleavage process of NOTCH proteins, inhibiting NOTCH protein expression by suppressing the NOTCH pathway.^172^ In a phase I trial, two ACC patients treated with the γ‐secretase inhibitor AL101 showed partial responses, with a duration of 8 months.^173^, ^174^ CB103, as a Pan‐NOTCH inhibitor, selectively blocks CSL–NICD interaction, leading to transcriptional downregulation of the oncogenic NOTCH pathway.^175^ Preliminary phase I clinical research has demonstrated its antitumor activity and safety, with 58% of patients experiencing disease stabilization and median PFS and OS of 2.5 and 18.4 months, respectively.

The effectiveness of NOTCH inhibitors in ACC is not yet fully elucidated, and reliable clinical data are limited. Several studies are ongoing, including a phase II trial (NCT03691207) based on AL101 for ACC patients with activating NOTCH mutations, and another phase II trial (NCT03422679) exploring CB103 in the treatment of advanced ACC patients.^176^, ^177^ In conclusion, the trial data on NOTCH inhibitors for ACC treatment are limited, and reliable conclusions are yet to be drawn. However, based on completed phase II studies, the treatment prospects appear promising.

Studies have shown that resistance to NOTCH inhibitors is related to the re‐expression of MYC through non‐NOTCH mechanisms, such as the use of alternative enhancers.^178^ Therefore, targeting downstream genes of the NOTCH pathway, like MYC, has emerged as a potential strategy for ACC treatment. Siu et al. used the PRMT5 inhibitor GSK3326595 to indirectly inhibit splicing and MYC expression, achieving partial responses in three out of 14 ACC patients.^179^ In recent years, direct MYC inhibitors, such as the omomyc mini‐protein, have also been developed, making this strategy increasingly feasible.^181^

*Other targeted therapies*. mTOR inhibitor rapamycin inhibits tumor proliferation by targeting the PI3K–Akt–mTOR pathway. A phase II trial (NCT01152840) with the mTOR inhibitor everolimus in progressive ACC patients showed no objective responses but had a 65.5% disease stabilization rate, with a median PFS of 11.2 months, indicating its promising therapeutic potential.^182^ Proteasome inhibitor bortezomib was also applied in a phase II trial with 25 ACC patients, reporting no objective responses but 17 cases of stable disease, with a median PFS of 8.5 months.^183^

Combination therapies, such as the triple combination of linsitinib (IGF1R inhibitor), gefitinib (EGFR inhibitor), and crizotinib (Alk and Met inhibitor), are currently in preclinical studies. While they have not shown significant clinical efficacy, they significantly reduced MYB expression, indicating potential clinical utility.^184^ Moreover, a phase II clinical trial is evaluating the combination of axitinib, a VEGFR inhibitor, and avelumab, an immune checkpoint inhibitor, for treating recurrent/metastatic ACC, with preliminary results suggesting its effectiveness. In ACC‐I, the significant overexpression of PAR, CHK1, and CHK2 suggests that PARP inhibitors might also be worth investigating.^185^ Additionally, BCL2 is markedly upregulated in ACC‐I, and it is co‐overexpressed with MYC and NICD1, indicating that apoptosis blockade could be a crucial process in the development of aggressive ACC. Preclinical testing has already shown that BCL2 inhibitors and GSIs have synergistic effects in other solid tumors, supporting this combined strategy.^186^, ^187^

Liu et al.^188^ have identified TROP2 as a potential therapeutic target for breast ACC, opening new avenues for targeted therapies in this specific subtype. Additionally, the oral CDK9 inhibitor KB‐0742 has shown a DCR of 53.8% in a cohort of 18 ACC patients, indicating its potential efficacy in managing ACC.^189^ Furthermore, TAK‐676, a novel STING agonist, is currently being evaluated in a clinical trial at Massachusetts General Hospital in Boston for patients with recurrent/metastatic ACC, aiming to assess its ability to activate the immune response against ACC.^190^

#### 5.3.2 Immunotherapy

ACC typically exhibits low immunogenicity, with histological examinations revealing minimal lymphocyte infiltration and rare expression of PD‐L1.^191^ Consequently, there are limited reports on therapeutic strategies targeting the immune mechanisms of ACC.^81^, ^101^ In the clinical trial KEYNOTE‐028, involving 26 patients with histologically advanced salivary gland cancer, including two with ACC, treated with pembrolizumab, the partial response rate was 12%, with a median duration of response of 4 months.^192^ Another clinical trial, NCT03087019, enrolled 20 patients with metastatic ACC. Considering that radiotherapy can increase the proportion of CD8+ lymphocytes and CD8+/FoxP3+ regulatory T cells in the ACC TME, enhancing responsiveness to immunotherapy, this trial also included a combination of pembrolizumab and radiotherapy, but no objective response was observed.^193^ Therefore, an effective immunotherapy regimen for ACC patients has not yet been identified.

In summary, recent translational efforts have been focused on exploiting these molecular insights to pioneer novel therapy approaches (Table 3). Molecularly targeted therapeutics, such as TKIs and MYB‐targeted strategies, have shown promise in preclinical studies. Immunotherapy, capitalizing on our growing understanding of the immune landscape in ACC, is also emerging as a potential treatment avenue. However, the immune milieu in ACC is characterized by a cold immune environment with low T‐cell infiltration and immunosuppressive features, challenging the efficacy of current immunotherapeutic strategies. Among all drug treatments, antiangiogenic therapies (VEGFR inhibitors) such as apatinib and axitinib have achieved the highest pathological response rates, proving to be the most effective drugs for treating ACC. New targeted therapies, such as NOTCH inhibitors and MYB inhibitors, are still in the early stages due to the relative lack of highly selective inhibition. However, based on current molecular mechanism studies of ACC, these therapies hold significant promise for future treatment strategies.

**TABLE 3:** Clinical trials on targeted therapy for ACC.

				Treatment efficacy		
Year	Trial number	Target	Drug	Response	Survival	References
2005		BCR‐ABL, c‐KIT, PDGFRA	Imatinib 400 mg daily	0% (0/4)		^194^
2005			Imatinib 800 mg daily for 2 months, if stable disease: P 80 mg/m^2^ every 3w + Imatinib 400 mg daily	0% (0/18)		^195^
2005			Imatinib 400 mg twice daily	0% (0/5)		^196^
2005			Imatinib 40 mg twice daily	0% (0/16)	PFS = 10w OS = 20w	^158^
2005			Imatinib 800 mg daily	0% (0/8)		^197^
2007			Imatinib 400 mg twice daily	12% (2/17)		^198^
2007			Imatinib 400 mg daily	0% (0/10)	PFS = 6 m	^157^
2016	NCT00859937	BCR‐ABL, SRC	Dasatinib 70 mg twice daily	2.5% (1/40)	PFS = 19.2 m	^199^
2016	NCT01703455	Ras–Raf, VEGFR	Sorafenib	11% (2/19)	PFS = 8.9 m OS = 26.4	^166^
2005		EGFR	Gefitinib 250 mg daily	0% (0/19)	PFS = 13w	^160^
2015	NCT00509002		Gefitinib 250 mg daily	0% (0/18)	PFS = 4.3 m	^200^
2007		EGFR, HER2	Lapatinib 1500 mg daily	0% (0/19)		^162^
2021	NCT02775370	VEGFR	Apatinib	46.2 (30/65)	PFS = 19.7 m	^169^
2016	NCT01558661		Axitinib (AG‐013736)	75.8% (25/33)	PFS = 5.7 m	^201^
2019			Axitinib 10 mg daily	Ongoing	Ongoing	^202^
2019	NCT03990571		Axitinib	18% (5/28)	PFS = 7.3 m OS = 16.6	^81^
2020	NCT02860936		Lenvatinib	11.5% (3/26)	PFS = 9.1 m OS = 27 m	^165^
2009			Cetuximab 400 mg/m^2^ loading dose, then 250 mg/m^2^ weekly.	0% (0/23)	OS = 15.8 m PFS = 3.4 m	^161^
2012	NCT00886132		Sunitinib 37.5 mg daily	0% (0/13)	PFS = 6 m	^203^
2017	NCT00581360	VEGF, FGF, PDGF	Dovitinib	8.3% (1/12)	PFS = 8.2 m	^204^
2011	NCT00077428	26S‐β	Bortezomib 1.3 mg/m^2^ days 1, 4, 8, 11 every 3 weeks. At progression: doxorubicin 20 mg/m^2^ days 1,8	0% (0/25)	OS = 21 m PFS = 6.4 m	^183^
2014	NCT01152840	mTOR	Everolimus 10 mg daily	0% (0/34)	PFS = 11.2 m	^182^
2015	NCT01065844	HIV protease	Nelfinavir 1250 mg twice daily	0% (0/15)	PFS = 5.5 m	^205^
2018	NCT03422679	NOTCH	CB‐103 daily	Ongoing	Ongoing	^175^
2018	NCT01778439		Brontictuzumab	16.7% (2/12)	PFS = 65d	^206^
2019	NCT03691207		AL101 4 mg weekly	Ongoing	Ongoing	^176^
2019	NCT03287427	MYB	TetMYB vaccine	Ongoing	Ongoing	^145^
2020	NCT03999684		ATRA	0% (0/18)	PFS = 3.2 m	^144^
2021	NCT03087019	PD‐1	Pembrolizumab	0% (0/20)	PFS = 4.5 m	^193^
2022	NCT04879849	STING	TAK‐676	Ongoing	Ongoing	^207^
2024	NCT06462183	MYB	RGT‐61159	Ongoing	Ongoing	^146^
2024	NCT06118086	CDK9	KB‐0742	Ongoing	Ongoing	
2024	NCT05775406	MDM2	KT‐253	0% (0/2)	Ongoing	^208^
2024	NCT03886831	NOTCH	PRT543	2% (1/56)	PFS = 5.9 m	^209^

Abbreviation: ATRA, all‐trans retinoic acid.

*Data sources*: https://clinicaltrials.gov/.

## 6 EMERGING RESEARCH AND FUTURE DIRECTIONS

The precision afforded by scRNA‐seq and integrated analyses of public databases has illuminated the convoluted cellular architecture of ACC, thereby sharpening the focus for future therapeutic interventions. Moving forward, a concerted effort is needed to validate the functional significance of the identified cellular subpopulations and molecular targets, ideally leading to more customized and efficacious treatments for ACC patients.

### 6.1 Advancing research models

Current basic research models for ACC primarily include cell lines and 3D models: patient‐derived organoid (PDO) and PDX models. While ACC cell lines such as ACC2 and SACC‐83 have been established, many have undergone significant genetic changes, and some are contaminated by nonhuman cells, limiting their accuracy.^210^ PDO models, developed by Lassche et al.,^211^ exhibit phenotypes consistent with the parent tissue and retain MYB (L1)/NFIB gene rearrangements, though their success rate is only 19%.^212^, ^213^ PDX models, created by Rose et al.,^214^ maintain the genomic changes of the primary tumor and have a higher success rate of 60%, but are more complex to produce.^215^, ^216^, ^217^, ^218^ To advance ACC research, it's crucial to develop cell lines with diverse genetic backgrounds and more stable 3D organoid models. Additionally, genetically engineered mouse models and orthotopic transplantation models, where ACC cells are implanted into the salivary glands of mice, could provide more robust platforms for studying ACC, addressing the limitations of current models.

### 6.2 Combining targeted therapies

Increasing evidence suggests that due to the significant variability among ACC patients and the heterogeneity of the tumor's single‐cell landscape, it is not wise to rely solely on a single drug for treatment. A promising strategy involves targeting personalized specific targets in patients and using inhibitors of compensatory targets in combination. For instance, combining NOTCH inhibitors with compensatory MYC inhibitors, using AXL and MET inhibitor cabozantinib with compensatory EGFR inhibitors, combining MEK inhibitors with compensatory p38 inhibitors, and using BCL2 inhibitors with GSIs or PRMT5 inhibitors. Several clinical trials are currently underway, which may be the most promising approach to overcoming ACC heterogeneity.

While challenges remain, particularly in translating these scientific insights into clinical improvements, the path forward is marked by an enhanced understanding of ACC's molecular diversity, and by the promise of therapeutic innovations tailored to target its intricate biological landscape. Continued research efforts, bolstered by ongoing advances in single‐cell technologies and integrative data analyses, are poised to significantly impact the management and prognosis of patients with ACC.

## AUTHOR CONTRIBUTIONS

*Haitang Yang and Feng Yao*: initiated the conceptualization of the paper. *Haitang Yang, Yunxuan Jia, and Yupeng Liu*: contributed to the writing of the main manuscript, providing detailed reviews and edits. *Yupeng Liu, Yunxuan Jia, and Haitang Yang*: were involved in figure creation. *Haitang Yang and Feng Yao*: provided comprehensive guidance throughout the process. All authors have read and approved the final manuscript.

## CONFLICT OF INTEREST STATEMENT

The authors declare that they have no conflict of interest.

## ETHICS STATEMENT

This study did not involve any experiments with human participants or animals performed by any of the authors. Ethical approval was not required for this study.

## Data Availability

Data sharing is not applicable to this article as no new data were created or analyzed in this study. All data supporting the findings of this study are included within the article
